# Mental health problems in tunisian military population

**DOI:** 10.1192/j.eurpsy.2023.1900

**Published:** 2023-07-19

**Authors:** D. Njah, H. Kefi, I. Bouzouita, A. Baatout, C. Bencheickh, S. Eddif, A. Oumaya

**Affiliations:** Psychiatry, military hospital, tunis, Tunisia

## Abstract

**Introduction:**

Military personnel can face unique risks and challenges to their mental health. High-stress situations, prolonged absences, and difficulty adjusting to civilian life can affect their mental health and hence develop psychiatric disorders , particularly major depressive disorder (MDD) and post-traumatic stress disorder( PTSD). That’s why searching for involved factors that could have an impact on these mental disorders or help predict them is crucial in the military population.

**Objectives:**

Our objectives were to describe the epidemiological profile of military patients followed in the psychiatric department of the military hospital of Tunis (MHT) and to identify the risk factors associated to psychiatric disorders in this population.

**Methods:**

This was a retrospective study conducted over a period of 4 weeks , in the psychiatry department of the MHT. We included in our study patients drawn at random at the outpatient clinics, all psychiatric disorders included. We analyzed the epidemiological characteristics of the patients as well as the risk factors with the SPSS software 26.0.

**Results:**

One hundred military patients were included in our study. The mean age of the patients was 38.74(±9.73) years, 93% of them were male, 86% had a high school education, 71% belonged to middle socioeconomic category, and 59% lived in the military barracks. The mean duration of service was 17.68(±9.22) years. Active military members were assigned to weapons jobs (45%), administrative (15%), technical (24%), transportation (8%), and health (6%) specialities.We found that MDD was the main psychiatric disorder found in 64% of the patients with a mean severity of 76.9%. Besides , administrative specialities were the most frequent source of MDD (73.3%), while transportation posts were the most common cause of the PTSD (12.5%). And finally weapons specialties were the most likely to cause adjustment disorders (13.3%). In addition, we found that a long military service duration was associated with a chronic evolution of all the mental disorders (p :0.002).

**Conclusions:**

The army is mostly affected by major depressive disorder . The position occupied by the patient seems to play a role in the type of the disorder . The seniority in the military service would be a risk factor for chronicity of the mental disorder.

**Disclosure of Interest:**

None Declared

